# Histological regression of gastrointestinal peritoneal metastases after systemic chemotherapy

**DOI:** 10.1515/pp-2021-0118

**Published:** 2021-07-15

**Authors:** Laura Toussaint, Hugo Teixeira Farinha, Jean-Luc Barras, Nicolas Demartines, Christine Sempoux, Martin Hübner

**Affiliations:** Department of Visceral Surgery, Lausanne University Hospital (CHUV), University of Lausanne (UNIL), Lausanne, Switzerland; Institute of Pathology, Lausanne University Hospital (CHUV), University of Lausanne (UNIL), Lausanne, Switzerland

**Keywords:** chemotherapy, peritoneal metastasis, peritoneal regression grading system (PRGS), PIPAC

## Abstract

**Objectives:**

Peritoneal metastases (PM) are relatively resistant to systemic chemotherapy, and data on histological response to therapy is rare. The aim of this study was to quantify the treatment response of PM after systemic chemotherapy.

**Methods:**

Retrospective monocentric cohort study of 47 consecutive patients with PM from gastrointestinal origin undergoing surgery (cytoreduction: CRS + Hyperthermic IntraPEritoneal Chemotherapy [HIPEC] or Pressurized IntraPeritoneal Aerosol Chemotherapy [PIPAC]) after prior systemic chemotherapy from 1.2015 to 3.2019. Tumor response was assessed using the 4-scale Peritoneal Regression Grading System (PRGS) (4: vital tumor to 1: complete response).

**Results:**

Patients had a median of 2 (range: 1–7) lines and 10 (3–39) cycles of prior systemic chemotherapy. A median of four biopsies (range: 3–8) was taken with a total of 196 analyzed specimens. Twenty-four biopsies (12%) showed no histological regression (PRGS4), while PRGS 3, two and one were diagnosed in 37 (19%), 39 (20%), and 69 (49%) specimens, respectively. A significant heterogeneity was found between peritoneal biopsies in 51% patients. PRGS correlated strongly with peritoneal spread (PCI, p<0.0001), and was improved in patients with more than nine cycles of systemic chemotherapy (p=0.04). Median survival was higher in patients with PRGS < 1.8 (Quartiles one and 2) than higher (Q3 and Q4), but the difference did not reach significance in this small cohort.

**Conclusions:**

PRGS is an objective too to describe histological response of PM of GI origin after systemic chemotherapy. This response differs significantly between patients, allowing to distinguish between chemosensitive and chemoresistant tumors.

## Introduction

As compared to liver metastasis, peritoneal metastases (PM) have a relatively limited response to systemic chemotherapy, and their prognosis remains poor in most disease entities [1]. In addition, evaluation of treatment response tends to be difficult, as many patients have no target lesions allowing evaluation according to RECIST criteria [2]. One interesting alternative is the assessment by histological response, and a 4-grade standardized evaluation system, the peritoneal regression grading system (PRGS) that was proposed and validated recently. The PRGS has been explicitly developed for taking into account specific characteristics of PM, such as their frequent mucinous character [3, 4]. Intraperitoneal treatment modalities like Heated Intra Peritoneal Chemotherapy (HIPEC) and Pressurized Intra Peritoneal Aerosol Chemotherapy (PIPAC) offer access to tumor biopsies in patients who, in the majority, received a systemic treatment previously [5, 6].

The aim of this study was to quantify the histological response of PM after previous systemic chemotherapy. Moreover, we aimed to determine a possible predictive value of PRGS after systemic chemotherapy. Our hypothesis was that a favorable PRGS would correlate with better overall survival.

## Materials and methods

This retrospective cohort study included non-selected patients admitted for intraperitoneal chemotherapy (PIPAC and HIPEC) after systemic chemotherapy for cancer of gasto-intestinal (GI) origin from January 2015 (start of PIPAC program in our department) to March 2019. The systematic use of PRGS for evaluating response of PM to intraperitoneal chemotherapy was started in our institution immediately after it became available in June 2016, and was used prospectively since then on a routine basis. In addition, the PRGS was retrospectively assessed for the patients treated between January 2015 and June 2016. Only patients with GI primary were considered for this analysis (n=47). Excluded were patients without previous chemotherapy, without histological sampling during surgery, missing PRGS assessment, or patients who refused to participate in the study (n=7). Only primary procedures were considered, and patients with previous intraperitoneal chemotherapy of any kind were excluded. In the case of PIPAC, only biopsies from the first procedure were selected for analysis. The Institutional Review Board of the CHUV University Hospital approved the study (CER-VD 2019-00747).

### Data management

Pertinent demographics, oncological and pathological data were retrieved from a prospectively maintained institutional database and entered in an *a priori* defined anonymized database containing the following variables: Demographics: age, gender, primary tumor origin, body mass index (kg/m^2^), ASA physical status classification score, serum tumor markers (CA 19-9 (kU/l), CEA (µg/l), CA-125 (kU/l)) and KRAS/HER_2_ amplification; Chemotherapy regimen, number of lines and cycles, PCI (Peritoneal Cancer Index) and PRGS scores (obtained during the first PIPAC or HIPEC).

### Surgical approach

PIPAC was performed by use of a two-trocar technique in a strictly standardized way [5, 7]. Cytoreductive surgery and HIPEC were performed through a midline laparotomy. In both approaches, laparoscopic or open, first step of the procedure was a systematic and complete exploration of the abdominal cavity with documentation of the Peritoneal cancer index (PCI). Biopsies were collected in suspect areas representing, whenever possible, at least four different areas of the abdomen. These were excisional biopsies for the open HIPEC cases and small samples taken with biopsy forceps during the laparoscopic PIPAC cases [4, 5]. Of note, biopsies were taken before surgical resection or delivery of intra-peritoneal treatment.

### Assessment of histological response

All peritoneal biopsies were assessed by a board-certified pathologist specialized in peritoneal cancer specimens. Tumor regression was evaluated using the Peritoneal Regression Grading Score (PRGS). PRGS discriminates four categories based on the presence of residual tumor cells and the extent of regression features, as described previously [4]. The PRGS was calculated as the mean of at least four biopsies from each abdominal quadrant, if technically possible [3, 4]. Also, the minimal (= the best regression) and the maximal (= the lesser regression) were documented.

### Predefined subgroup analyses

Predefined stratifications were primary tumor types, number of lines, and cycles of previous chemotherapy and treatment modality (PIPAC vs. HIPEC). Patients were grouped into two entities: (1) upper gastrointestinal tumors (UGI) group including patients with gastric cancer and (2) lower gastrointestinal tumors (LGI) including patients with PM from rectal, colic, and small bowel origin.

### Statistics and analysis

PRGS was presented as mean ± SD for patients having four biopsies at least, and in addition, the highest and lowest grading was reported [4]. Continuous variables were presented as mean with standard deviation (SD) or median with range or interquartile range (IQR) for skewed data. Categorical variables were reported as frequencies (%) and compared with the chi-square test. Depending on the normality of distribution, Student’s *t*-test and Mann–Whitney U test or Wilcoxon signed ranked test were used for float comparisons. Statistical correlations were tested by use of Pearson’s rank correlation. A level of 0.05 was considered statistically significant. Statistical analyses were performed, and figures were produced with SPSS v20 software (Chicago, IL, USA), GraphPad Prism 7 (GraphPad Software, Inc., La Jolla, CA, USA), Python, NumPy, Pandas, and Seaborne (Anaconda, Berlin, Germany).

## Results

Forty-seven consecutive patients were included for the analysis: 47 patients were treated with PIPAC and six with HIPEC. Median follow-up after surgery (HIPEC or first PIPAC) was 27 months (IQR 17-36). 17 patients (36.1%) died within this period of time. The study cohort was heterogeneous with regards to primary tumor and previous treatments, as detailed in Tables 1 and 2.

**Table 1:** Patients baseline demographics.

	All patients (n=47)	LGI (n=37)	UGI (n=10)	p-Value
Median age (IQR)	60 (52–69)	61 (52–70)	57 (51–67)	0.500
Gender, male	22 (47%)	16 (43%)	6 (66%)	0.346
ASA score				0.852
2	34 (66%)	27 (73%)	7 (70%)	
3	16 (34%)	10 (27%)	3(30%)	
Median PCI (IQR)	12 (4–24)	13 (4–24)	10 (5–29)	0.832
PIPAC (%)	41 (87%)	31 (76%)	10 (24%)	0.556
CRS + HIPEC (%)	6 (13%)	6 (100%)	0	
Prior chemotherapy received				
Median lines (range)	2 (1–7)	2 (1–7)	1 (1–2)	0.129
Median cycles (range)	11 (3–39)	11 (3–39)	12 (3–12)	0.471

Median (IQR or range) or number (%) as appropriate. Statistical significance (p<0.05) is highlighted in italics. ASA, American Association of Anesthesiologists physical status classification system; UGI, upper gastroIntestinal tumour; LGI, lower gastroIntestinal tumour; PIPAC, Pressurized IntraPreritoneal Chemotherapy; CRS + HIPEC, CytoReductive Surgery + Hypertermic IntraPeritoneal Chemotherapy.

**Table 2:** Regimen used for the last line of prior chemotherapy.

	Number of patients	Number of cycles (median)
FOLFOX	8	9
FOLFOX + bevacizumab	6	6
FOLFOX + cetuximab	5	7
FOLFIRI	3	12
FOLFIRI + bevacizumab	9	7
FOLFIRI + cetuximab	8	6
Other regimens/unknown	8	–
Total	47	–

FOLFOX leucovorin + fluorouracil, FOLFIRI leucovorin + fluorouracil + irinotecan. Other: FOLFIRINOX (leucovorin + fluorouracil + irinotecan + oxaliplatine), Panitumab, Eporubicine, Capécitabine, Docetaxel, Premetrexed, Carboplatine

### Number of biopsies and variability

Overall, a median of four biopsies (range: 3–8) was taken with a total of 196 analysed specimens. Disrcepant PRGS values for the different tumor biopsies in the same patient were documented for 24 out of 47 patients (51%).

### Macroscopic (PCI) and microscopic (PRGS) assessments

**Figure 1:**
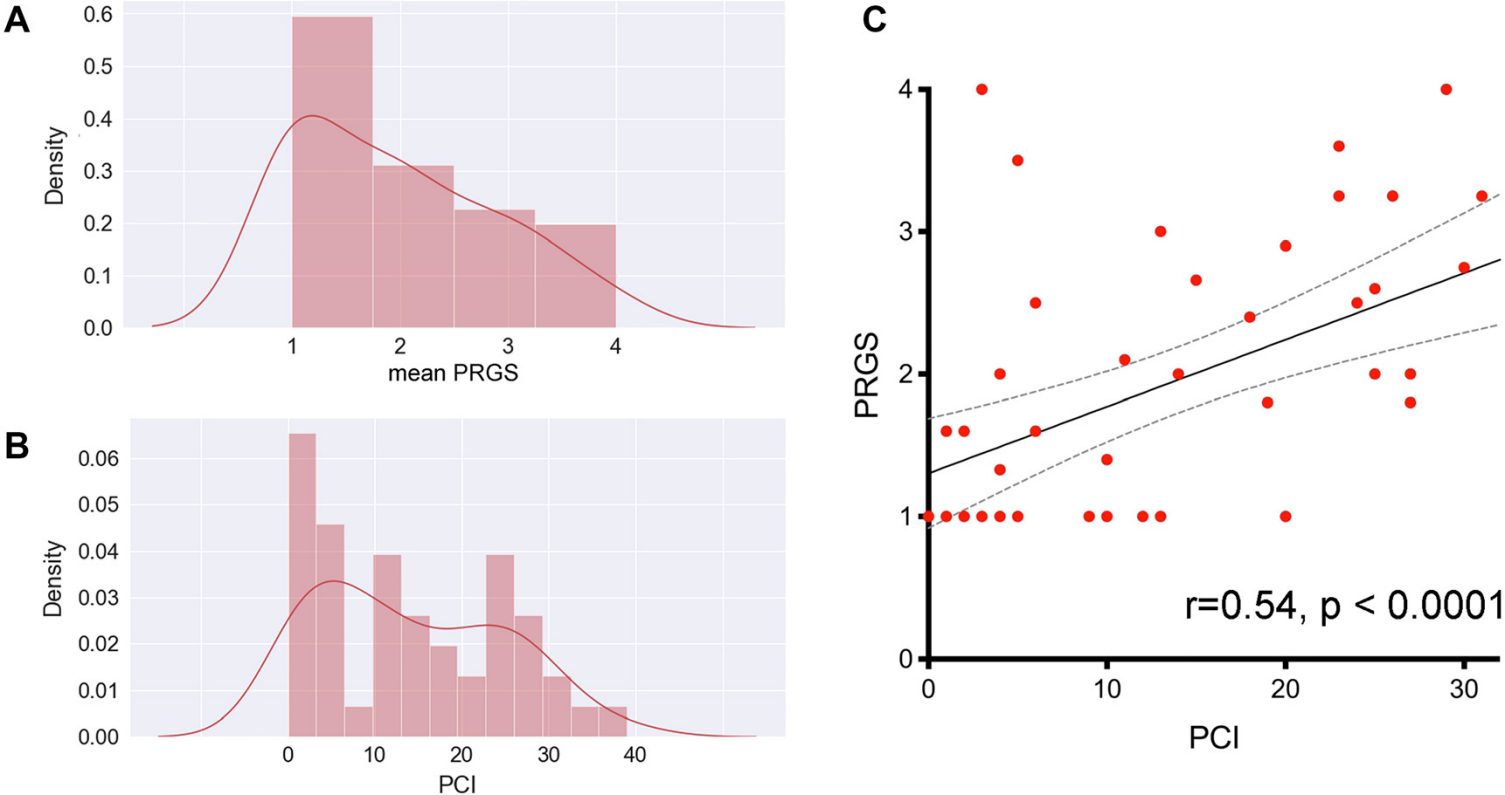
It this cohort of patients, the peritoneal regression grading score (PRGS, panel A) has a distribution similar to the peritoneal cancer index (PCI, panel B), which suggests a good concordance between microscopic (PRGS) and macroscopic (PCI) assessments of disease aggressivity. This concordance was confirmed by plotting the PCI against the PRGS (panel C). This correlation is highly significant without regard to the intensity and nature of the chemotherapy regimen received by individual patients.

Median PRGS was 1.8 (IQR 1.0-2.63) for the entire cohort. Median PCI was 12 (IQR 4-24) PCI and mean PRGS correlated strongly to each other (p<0.0001 ϱ = −0.540), showing an association between advanced disease extent and low histological regression (Figure 1). In gastric cancer patients (n=10), there was no statistical difference in PRGS between diffuse, intestinal, and intermediate histologies according to Lauren’s classification (Supplementary Material 1). No statistically significant differences of PRGS were measured between different UGI vs. LGI cancers or between different primaries.

### Tumor response to chemotherapy, objective histological regression

**Figure 2:**
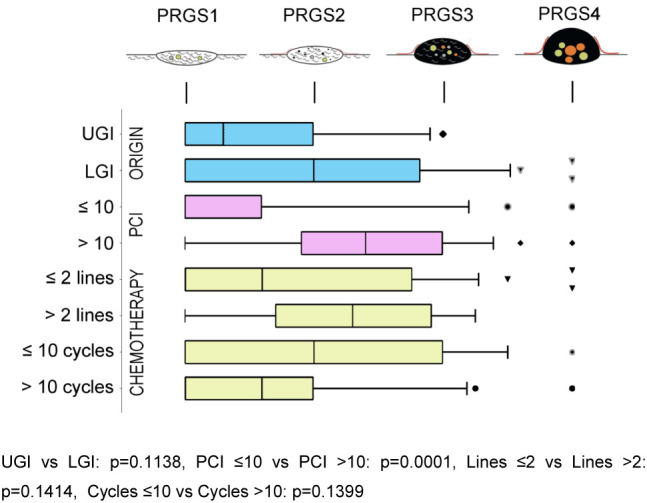
Sensitivity analysis of histological regression (PRGS) of peritoneal cancer after systemic chemotherapy. The horizontal box plots illustrate the PRGS stratified by tumor origin, PCI, previous chemotherapy lines, and cycles. PRGS1 = complete regression with the absence of tumor cells; PRGS2 = major regression features with only a few residual tumor cells; PRGS3 = minor regression with a predominance of residual tumor cells and few regressive features; PRGS4 = response. PRGS: median, 10, and 90 percentile with outlier’s data. LGI: Lower gastrointestinal tract; UGI: Upper gastrointestinal tract. The various symbols (outlier’s) represented are automatically generated by the program (GraphPad Prism 7). They are different in order to avoid confusing the lines.

Twenty-four peritoneal biopsies (12%) showed no histological regression (PRGS4), while PRGS 3, two or 1 (complete regression) was diagnosed in 37 (19%), 39 (20%), and 69 (49%) specimens, respectively. The overall grade of regression did not corelate with the number of chemotherapy lines, or cycles received previously. However, in a subgroup of patients treated with 10 and more chemotherapy cycles before surgery, a complete histological regression (PRGS 1) was documented more frequently than in patients treated with fewer cycles (p=0.04). Interestingly, histological response to Oxaliplatin correlated well with the increasing number of cycles p=0.02 ϱ = −0.381. Figure 2 summarizes the sensitivity analysis performed by tumor origin, the extent of peritoneal disease, and previous chemotherapy (number of lines, number of cycles received).

### PRGS and overall survival

**Figure 3:**
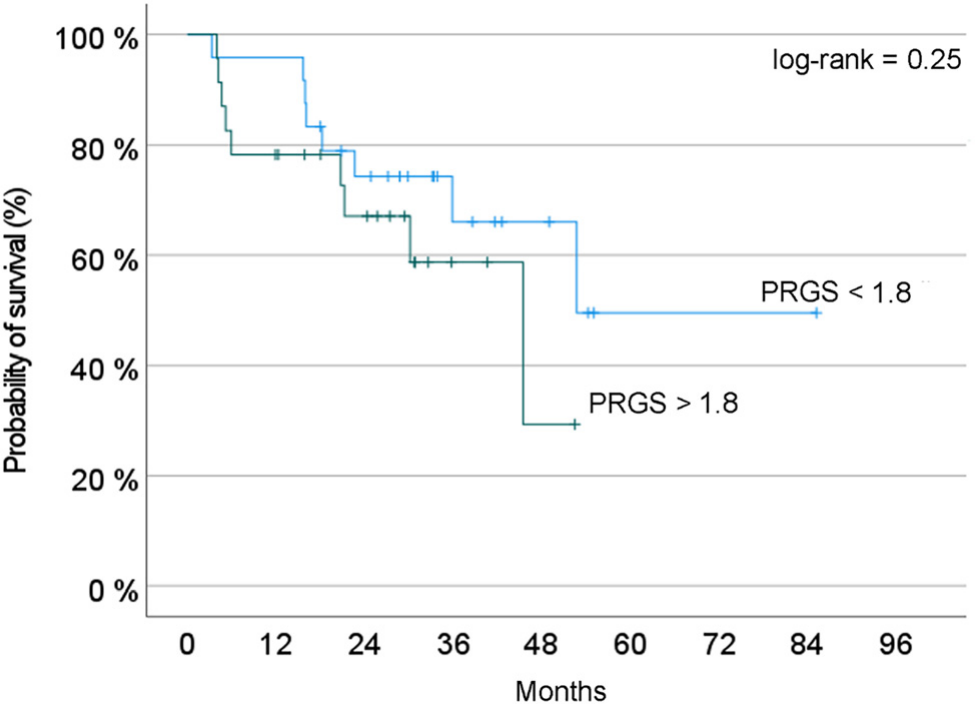
Probability of overall survival depending on PRGS. Two groups of patients are compared: Blue curve = patients with a PRGS inferior to the median value of the cohort; red curve: Patients with a PRGS superior to this value, suggesting a poorer prognosis. In this small cohort of patients, the difference observed did not reach statistical significance (log-rank test, p=0.25).

We hypothesized that a favorable PRGS would correlate with better overall survival. Considering the relatively small size of our cohort, we pooled the patients with an unfavorable mean PRGS (Quartiles three and 4) vs those with high or complete histological regression (Quartiles one and 2). Figure 3 shows the probability of overall survival, estimated with the Kaplan-Meyer method. This curve suggests a predictive value of PRGS for overall survival. However, this difference did not reach statistical significance (log-rank, p=0.25).

## Discussion

The Peritoneal Regression Grading Score (PRGS) was initially developed to quantify PM’s histological response to palliative intraperitoneal chemotherapy [8]. This study demonstrated that PRGS can also be used for measuring the objective response of PM to systemic, intravenous chemotherapy.

Several tumor regression systems (TRGs) have been proposed to quantify the tumor response to neoadjuvant chemotherapy, in particular in oesogastric [9], ovarian [10] and rectal [11, 12] cancer. A tumor regression grading system was also established for colorectal liver metastases [13]. The PRGS was the first score developed specifically for PM, taking into account specific features such as their frequent mucinous nature [3]. A generic, unique score for assessing histological tumor response to chemotherapy in PM makes sense because of the clinical impact of histological response to therapy and because the organ of metastasis (peritoneum) is the same [3]. The PRGS has been the object of a multi-institutional validation study [4] and is now diffusing into clinical practice [8, 14], [15], [16], [17], [18], [19], [20]. The PRGS is increasingly used as secondary [21], [22], [23], [24], or even as primary outcome criteria [25] in clinical studies on PM.

In this study, the assessment of tumor activity using the PRGS correlated well with the macroscopic tumor spread, measured as the PCI. This correlation suggests an association between advanced disease extent and poor histological regression. PRGS does not appear to be affected with the number of previous cycles and lines or PM origin. In view of our small group of patients, it is hazardous to conclude that these three variables have no effect on the PRGS.

To our knowledge, this has not been shown before and could be an indirect validation of PRGS. The PCI is widely accepted in the oncological community because it has a prognostic value [26] and because it can be used to determine the surgical resectability of PM [27]. However, the PCI measures only the number and the size of tumor nodes throughout the peritoneal cavity without giving information on the number of viable tumor cells within these tumor deposits. The PRGS delivers additional information on the vitality of the tumor nodes. This information might be used, for example, to refine the indication to cytoreductive surgery by excluding patients with vital, highly aggressive tumors after neaodjuvant treatment.

This study showed that PRGS values were discrepant in 51% patients, documenting different grades of tumor activity at different intraabdominal localizations. Thus, the PRGS demonstrated different morphology of PM simultaneously at various sites as a sign of tumor heterogeneity. As a consequence, multiple tumor biopsies are needed to obtain reliable information on the activity of the peritoneal disease. There is currently no evidence whether to consider the highest (worst) or the mean PRGS. Selecting the highest PRGS would follow the Union for International Cancer Control (UICC) recommendation for tumor grading (G1 to G3) [28]. However, selecting the highest PRGS would imply the loss of up to 75% of the information, a highly debatable option in data science. Whereas most groups are using the mean PRGS as a measure, the present recommendations suggest reporting both the highest and the mean PRGS [3, 4].

From the clinical perspective, the different PRGS values in individual patients documented variable degrees of response to systemic chemotherapy. Such variability is likely to be explained by the emergence of multidrug resistance by clonal selection under therapy. However, knowledge about PM’s clinical behavior or molecular patterns is scarce compared to parenchamytous metastasis. Recently, the heterogeneity of PM was highlighted in patients with colorectal cancer: in these patients, recurrent peritoneal metastasis after radical treatment represented a more aggressive subset [29]. The next step is now to generate molecular profiles of PM with the hope of identifying patterns with clinical significance. For example, it might be possible to identify patients with chemoresistant tumors who might not benefit from cytoreductive surgery [30] or adapt the chemotherapy regimen based on objective histological tumor response. The utility of next-generation sequencing to detect cancer-related mutations in peritoneal biopsies and peritoneal fluid after systemic chemotherapy and PIPAC treatment has been recently demonstrated [18]. Dynamic changes of tumor gene expression during PIPAC in women with PM have shown prognostic significance [31]. Thus, complementing PRGS with molecular information could pave the way for individualized therapy of PM patients.

We hypothesized that a favorable mean PRGS would correlate with better overall survival. The rationale for such hypothesis is strong since tumor with no malignant cells detected in the histology (PRGS1) are indeed expected to be less aggressive than highly vital tumors (PRGS 4). In a mouse model of PM from colorectal cancer, PRGS was a good measure of histological regression and was correlated with the efficacy of chemotherapy [32]. In clinical setting, a combined progression index based on PRGS and peritoneal CPI+ was an independent predictor of worse prognosis for overall survival (HR = 5.24]), and progression-free survival (HR = 4.41) [33]. As expected, in this small cohort, the patients survival with a low (= favorable) PRGS was superior to the patients with a high PRGS. Since our study was not powered and had only exploratory value, this encouraging finding should be interpreted with caution.

In conclusion, PRGS appears to be promising to assess treatment response in PM. The histological PRGS correlates with the macroscopical PCI, suggesting an association between advanced disease extent and poor histological regression. The PRGS highlights the phenotypic heterogeneity of PM within an individual patient. The PRGS can assess the tumor response not only to intraperitoneal but also to systemic chemotherapy. The baseline needs to be considered when evaluating intraperitoneal and/or systemic treatment’s potential incremental benefit. The PRGS might deliver important prognostic or even predictive information. However, the links between histological regression, molecular patterns, chemoresistance, and PM prognosis remain largely unclear and should be investigated in proper clinicopathological studies.
