# Supplementary figures and images for: Histological regression of gastrointestinal peritoneal metastases after systemic chemotherapy
Source: Pleura Peritoneum. 2021 Jul 15;6(3):113–9. doi: 10.1515/pp-2021-0118 (PMC8482450; doi:10.1515/pp-2021-0118)

**SUPPL MATERIAL 1**
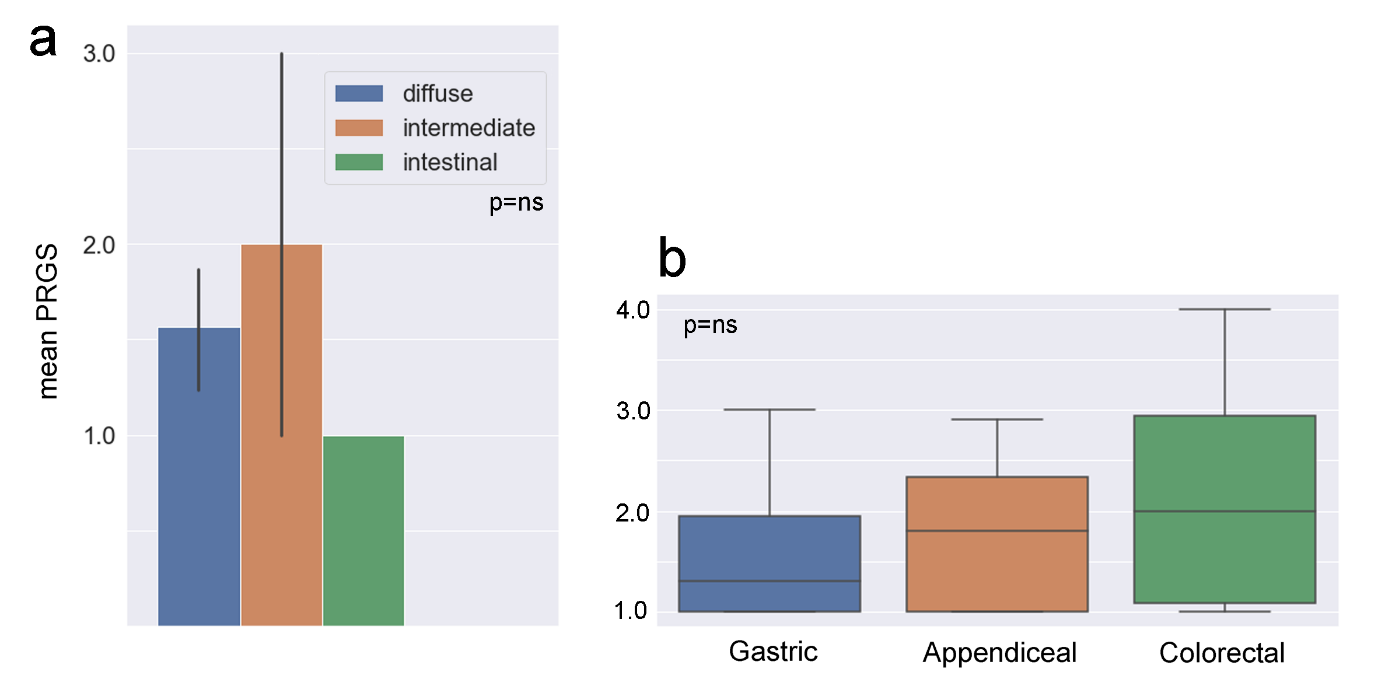

Supplement: Supplementary file 1 [file pp-06-20210118-s001.docx]
